# Supplementary figures and images for: Defense against parasites covaries with reproductive timing, not with resistance
Source: PLoS Pathog. 2026 Jul 9;22(7):e1014388. doi: 10.1371/journal.ppat.1014388 (PMC13367896; doi:10.1371/journal.ppat.1014388)

A

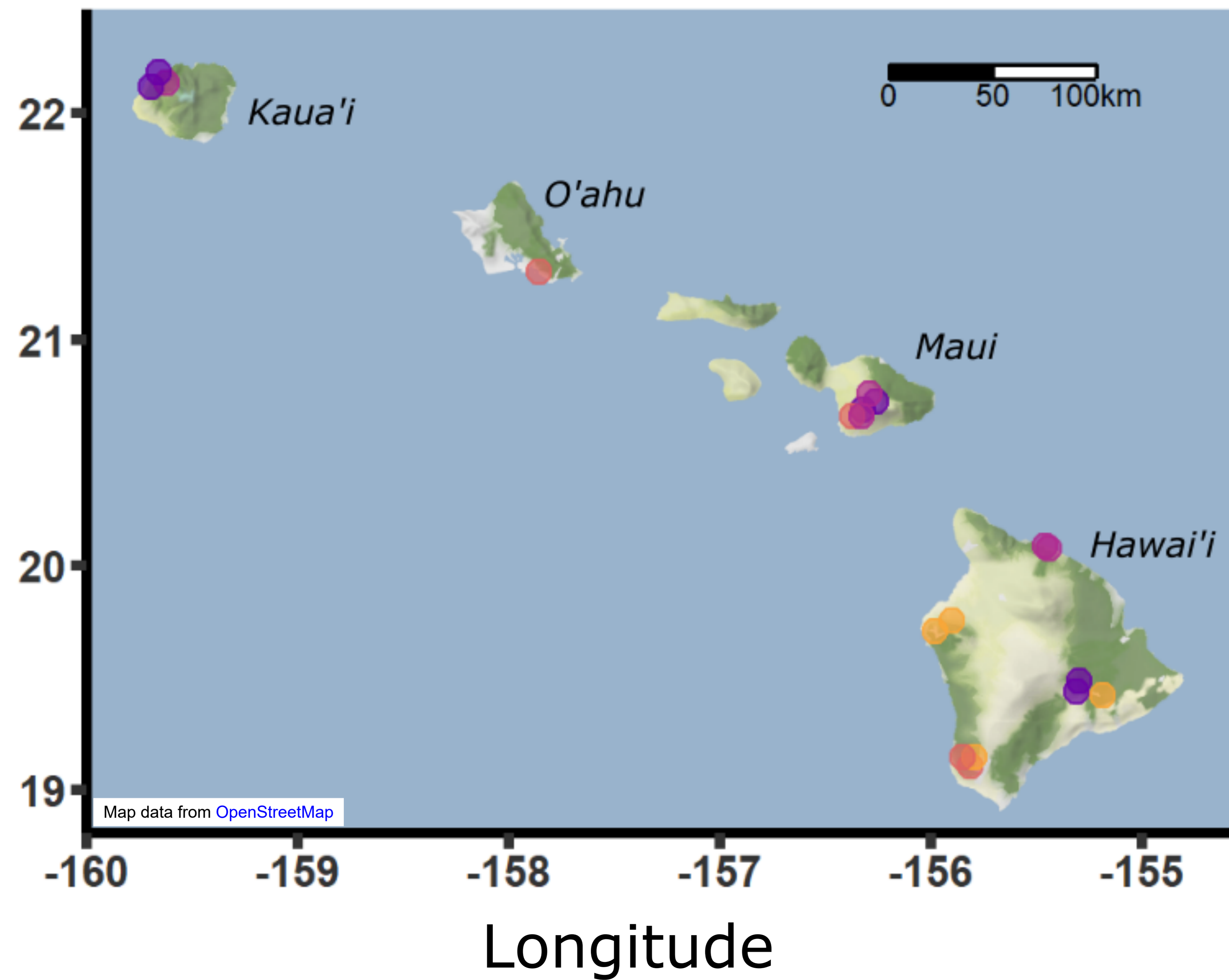

B

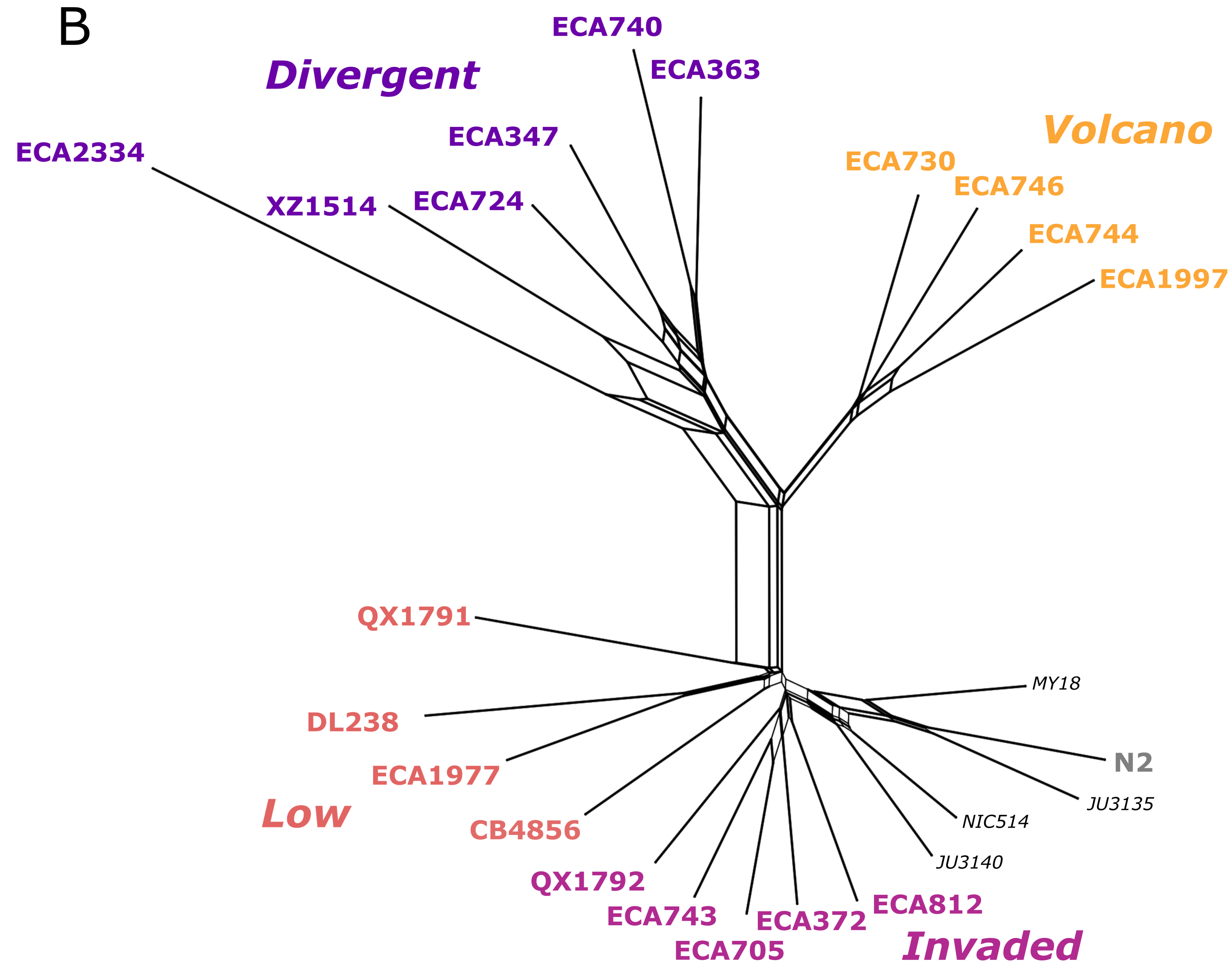

Supplement: S1 Fig — A shows the sampling locations of the 19 Hawaiian strains. Points are jittered to reduce overlap. The 20th strain, N2, is not from Hawaii and thus is not displayed on the map. The map was generated using the package ggmap [108]. The map tiles are by Stamen Design, Stadia Maps, and OpenMapTiles under CC BY 4.0. Map data are from OpenStreetMap, under ODbL. B shows a neighbor-joining network of the 20 strains included in this study, plus four non-Hawaiian strains (in italics) that were not included in this study but are shown for added phylogenetic context. Colors of points in A and taxa in B correspond to the four relatedness clusters (i.e., group: Divergent, Volcano, Invaded, and Low). The neighbor-joining network was generated using [47]’s Fig 5 VCF dataset as the base variant call set. Three strains not included in that dataset (ECA1997, ECA1977, ECA2334) were downloaded from CaeNDR as VCF variant data and filtered to retain comparable sites. The additional strains were harmonized with [47]’s call set to generate a combined SNP matrix for the selected taxa. The merged VCF was converted to a NEXUS-formatted SNP alignment using vcf2phylip.py and then used to generate the neighbor-joining network in SplitsTree4 [109]. Table A in S1 Text provides further details on the host strains. (PDF) [file ppat.1014388.s001.pdf]

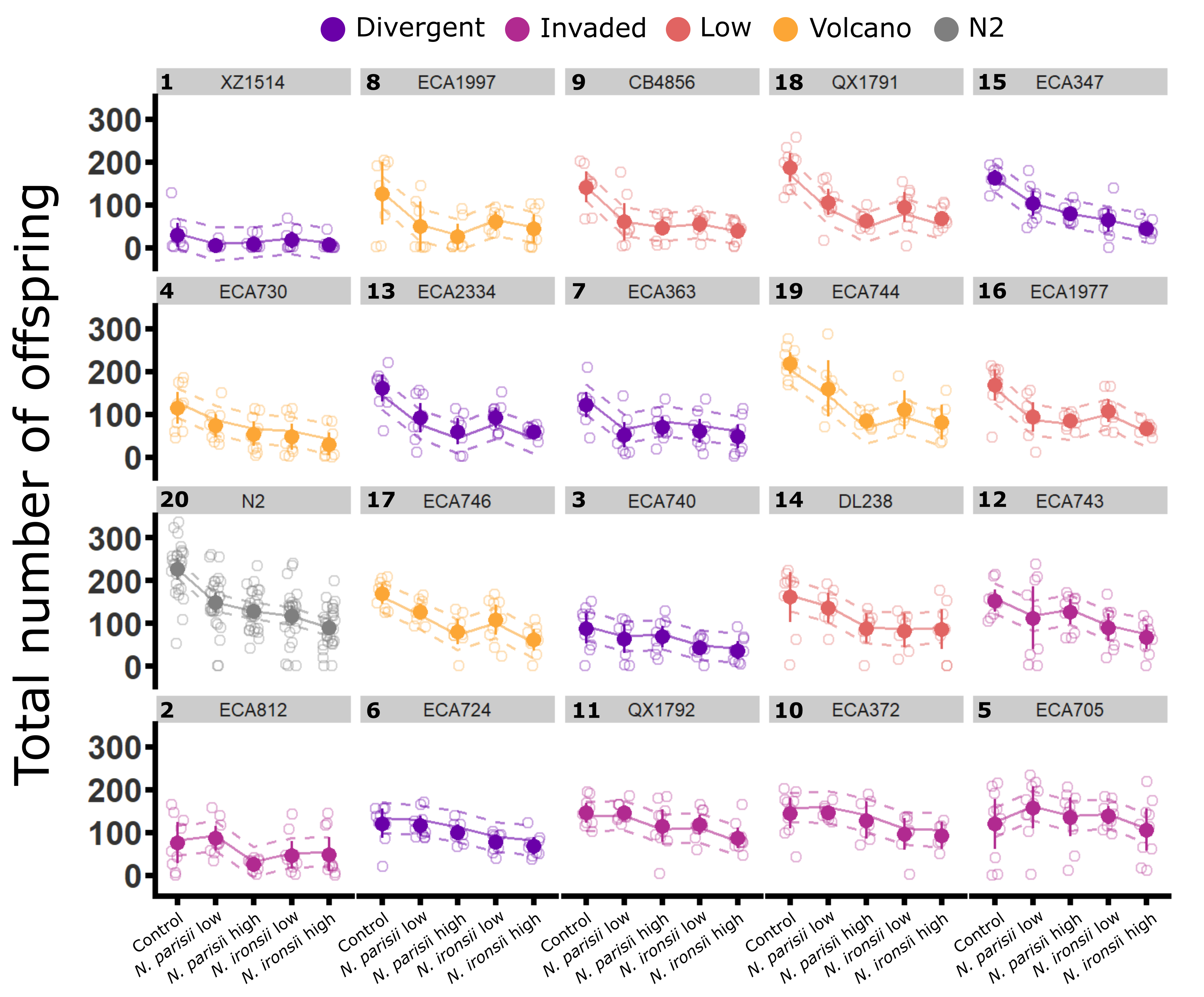

Supplement: S2 Fig — The total number of offspring per host is shown for each of the five treatments, faceted by host strain. Points show raw data for total fecundity, while the lines show estimated marginal means from the full linear mixed effects model in Table F in S1 Text. For the raw data, unfilled points show the total number of offspring for individual hermaphrodites, and filled points show the mean. Error bars show 95% confidence intervals. For model estimates, solid lines indicate the mean and dashed lines the 95% confidence interval. Host strains are arrayed from top left to bottom right in order of increasing overall defense against Nematocida, as shown in Fig 2a, and they are colored according to host group, with the host strain N2 in gray. Host strains are indicated by both their strain name and numeric ID. (PNG) [file ppat.1014388.s002.png]

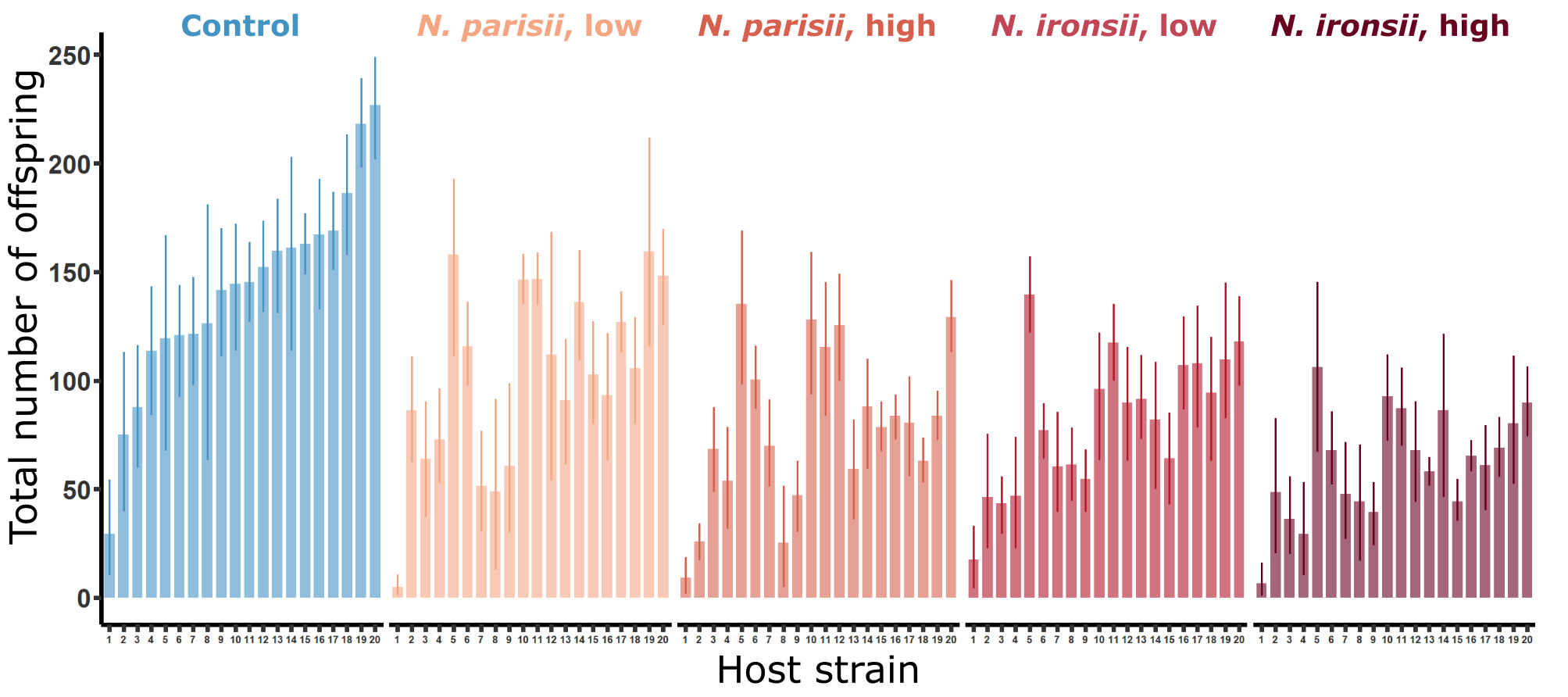

Supplement: S3 Fig — Bars show the mean number of offspring per host, with 95% confidence intervals, for each host strain. Within a treatment, host strains are ordered from left to right by increasing fecundity in baseline control conditions. Host strains are indicated by their numeric ID (Table A in S1 Text, Fig 1). (PNG) [file ppat.1014388.s003.png]

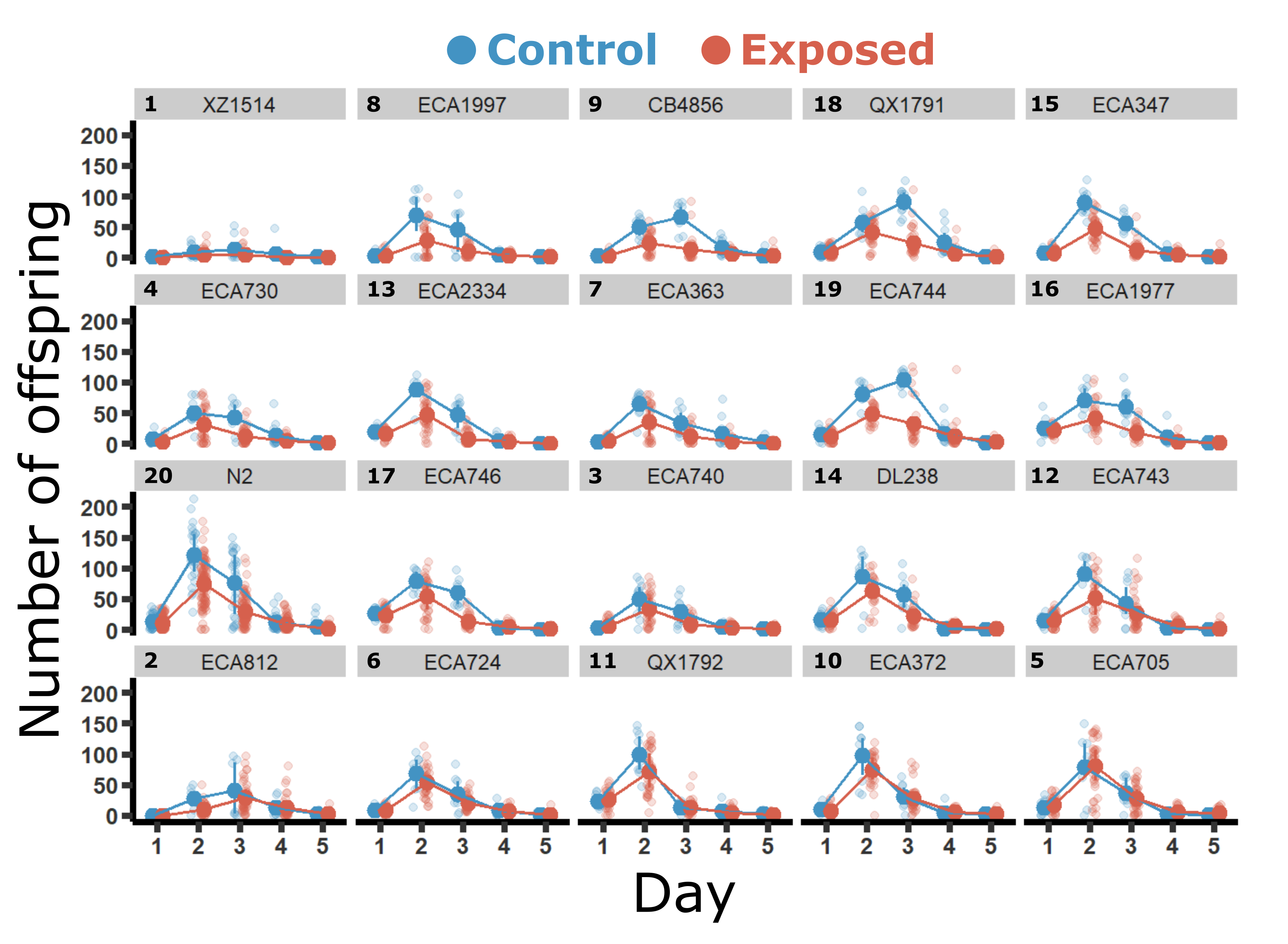

Supplement: S4 Fig — As in Fig 4a and 4b solid points indicate the mean number of offspring per host per day, and shaded points show raw data for individual hermaphrodites. Error bars show the interquartile ranges of the data. Blue denotes hosts in the control treatment, and red denotes hosts in exposed conditions, representing all four treatments. Host strains are arrayed from top left to bottom right in order of increasing overall defense against Nematocida, as shown in Fig 2a. Host strains are indicated by both their strain name and numeric ID. (PNG) [file ppat.1014388.s004.png]

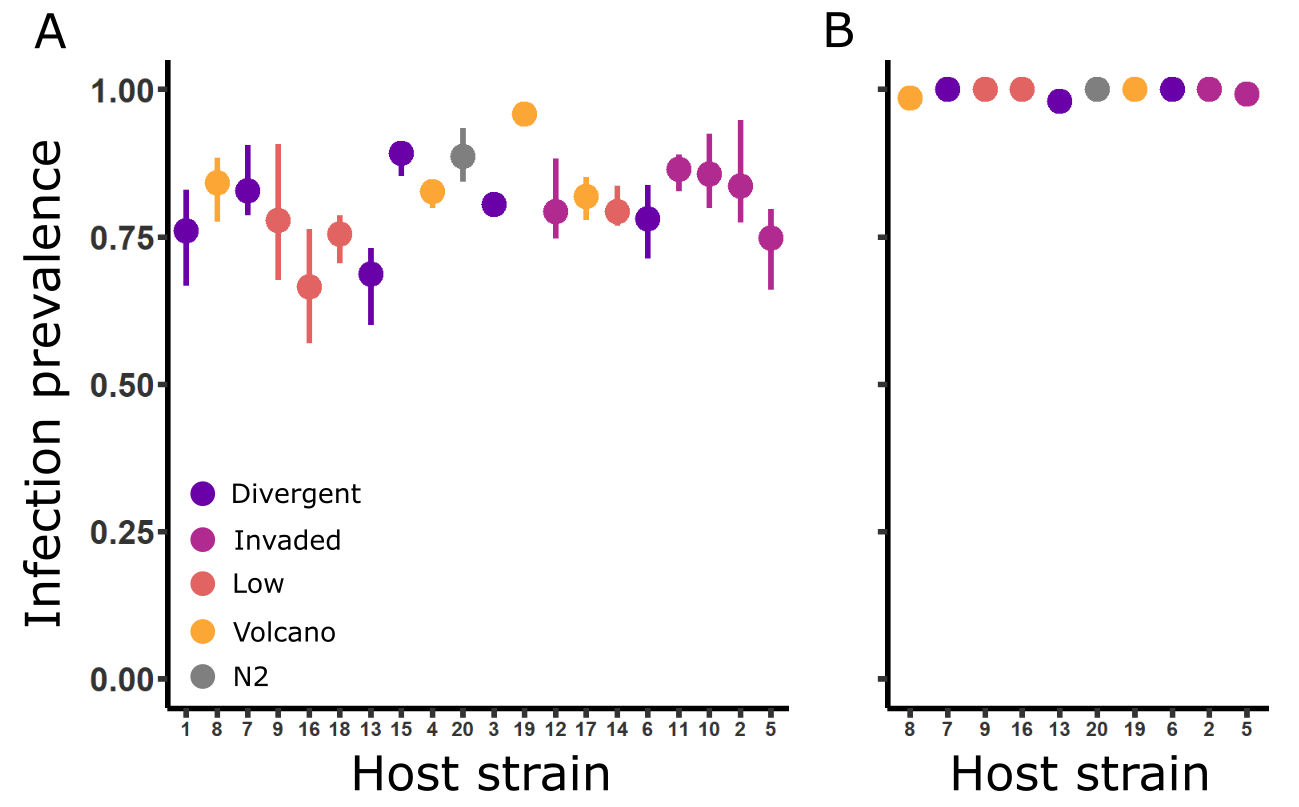

Supplement: S5 Fig — Circles indicate the mean prevalence of infection across replicates, and error bars show interquartile ranges of the data. Host strains are arrayed along the x-axis in order of increasing defense against a low dose of N. parisii, as shown in Fig 3b, top left, and they are colored according to host group, with the host strain N2 in gray. Host strains are indicated by their numeric ID. (PNG) [file ppat.1014388.s005.png]

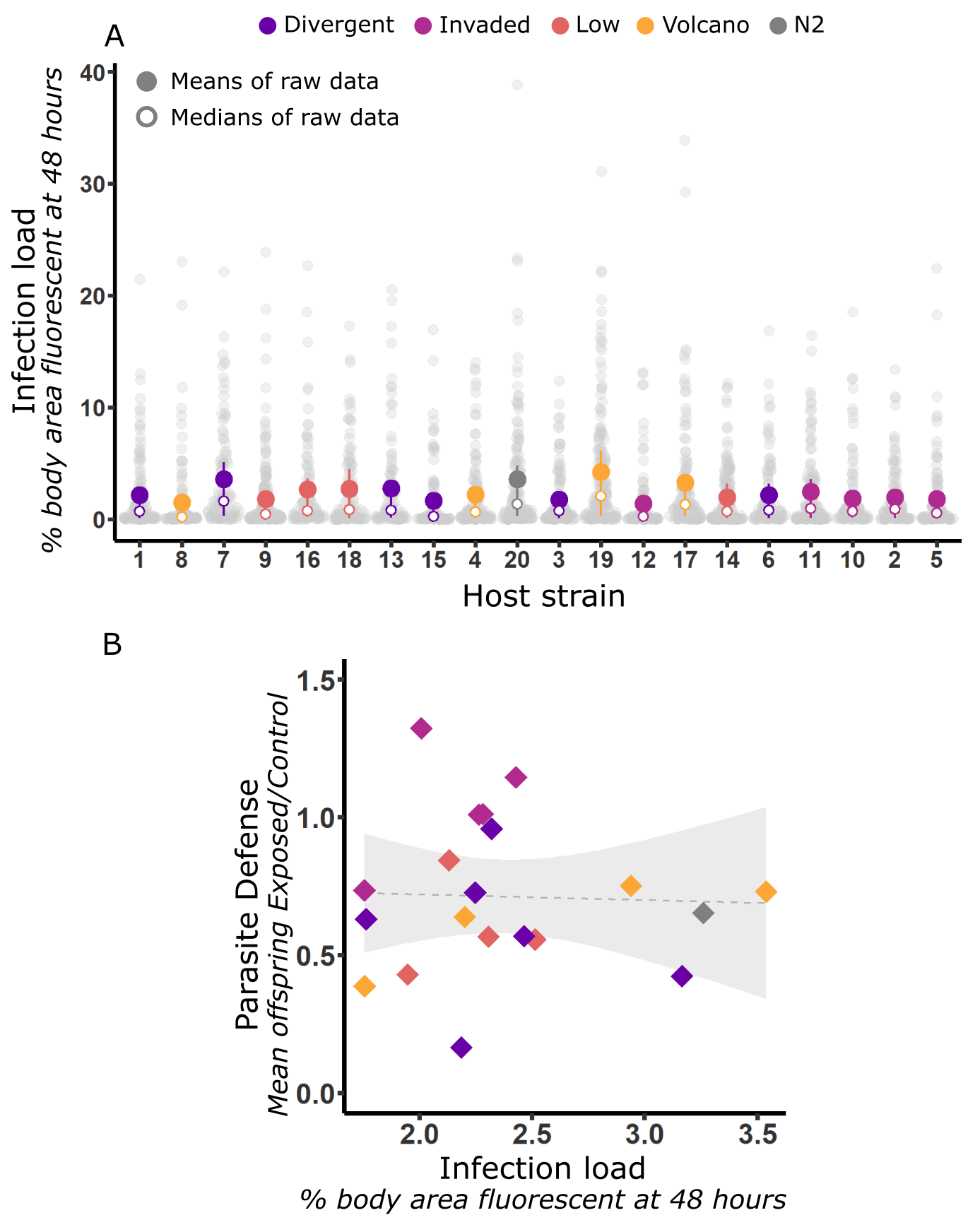

Supplement: S6 Fig — A presents infection load at 48 hours, measured as the percent of body area fluorescent in 2D images of hosts. Higher infection load is interpreted as lower resistance. Gray dots show infection load of individual hosts. Filled circles show means of the raw data plus interquartile ranges. Open circles show medians of the raw data. Host strains are arrayed along the x-axis in order of increasing defense against a low dose of N. parisii, as shown in Fig 3b, top left, and they are colored according to host group, with the host strain N2 in gray. Host strains are indicated by their numeric ID. B shows the relationship between infection load at 48 hours and defense. Increasing values on the x-axis indicate decreasing resistance. Each point represents a host strain, colored by host group. Defense is given for the response to a low dose of N. parisii, as in Fig 3b, top left. For load, diamonds show the means of model predictions for replicates from the conditional model in Table P in S1 Text. (PNG) [file ppat.1014388.s006.png]

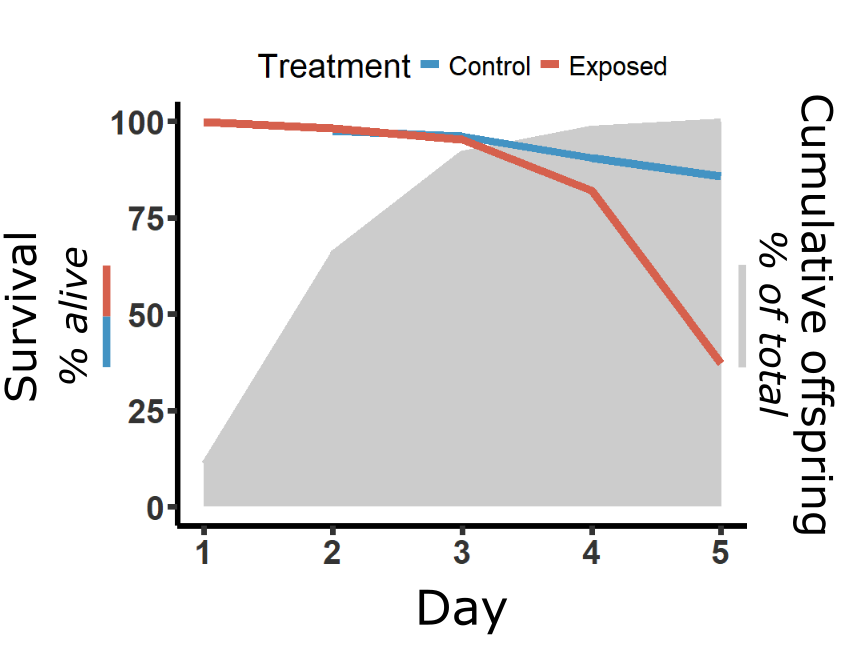

Supplement: S7 Fig — Lines show the percentage of hosts alive during the fitness assay, in control (blue) and exposed (red) conditions. The Control group represents data from 209 hosts across 20 strains, while the Exposed group represents data from 819 hosts from 20 strains and four exposure treatments. Gray shaded area shows the cumulative offspring production through time across all hosts; this is represented as the cumulative percent of total offspring produced after a given day of reproduction. Survival methods and results are presented in S2 Text. (PNG) [file ppat.1014388.s007.png]
