# Supplementary material for: Defense against parasites covaries with reproductive timing, not with resistance
Source: PLoS Pathog. 2026 Jul 9;22(7):e1014388. doi: 10.1371/journal.ppat.1014388 (PMC13367896; doi:10.1371/journal.ppat.1014388)
Supplement: S1 Text — (PDF) [file ppat.1014388.s008.pdf]

S1 TEXT

SUPPLEMENTAL TABLES FOR

Defense against parasites covaries with reproductive timing, not with resistance

Amanda K Gibson, Linyao Peng, Tessa Batterton, Neha Channamraju, Victoria Feist, Sarah Hesse, Anne N Janisch, and Hongyi Shui

I. Table A: Host strains
II. Table B: Details of spore preps and doses for each parasite species
III. Table C: Number of hermaphrodite hosts per strain and treatment in the fitness assay.
IV. Table D: Statistical analysis of total fecundity in control and exposed conditions across host strains
V. Table E: Statistical analysis of total fecundity in control and exposed conditions across host group
VI. Table F: Statistical analysis of total fecundity across treatments
VII. Table G: Statistical analysis of change in total fecundity from control for each exposure treatment
VIII. Table H: Statistical analysis of total fecundity across exposed treatments
IX. Table I: Correlation of defense across exposure treatments
X. Table J: Statistical analysis of daily fecundity by host strain in the control treatment
XI. Table K: Statistical analysis of daily fecundity by host group in the control treatment
XII. Table L: Statistical analysis of daily fecundity in control and exposed conditions
XIII. Table M: Linear regression of reproductive timing and defense against parasites
XIV. Table N: Linear regression of fecundity and defense against parasites
XV. Table O: Replication structure for resistance assays
XVI. Table P: Statistical analysis of infection prevalence and load at 48 hours.
XVII. Table Q: Statistical analysis of infection prevalence and load by group at 48 hours.
XVIII. Table R: Statistical analysis of infection load at 72 hours.
XIX. Table T: Statistical analysis of infection load by group at 72 hours.
XX. Table U: Linear regression of resistance and defense against parasites

Table A: Host strains

Strain/isotype	Group	Latitude	Longitude	Island	ID
CB4856	Low	21.330	-157.860	O'ahu	9
DL238	Low	19.110	-155.810	Hawai'i**	14
ECA1977	Low*	19.120	-155.820	Hawai'i	16
QX1791	Low	20.634	-156.394	Maui	18
ECA372	Invaded	22.122	-159.664	Kaua'i	10
ECA705	Invaded	20.039	-155.439	Hawai'i	5
ECA743	Invaded	20.742	-156.323	Maui	12
ECA812	Invaded	20.040	-155.442	Hawai'i	2
QX1792	Invaded	20.706	-156.355	Maui	11
ECA2334	Divergent*	22.133	-159.651	Kaua'i	13
ECA347	Divergent	19.439	-155.304	Hawai'i	15
ECA363	Divergent	20.724	-156.304	Maui	7
ECA724	Divergent	19.439	-155.304	Hawai'i	6
ECA740	Divergent	20.670	-156.339	Maui	3
XZ1514	Divergent	22.149	-159.668	Kaua'i	1
ECA1997	Volcano*	19.720	-155.949	Hawai'i	8
ECA730	Volcano	19.424	-155.222	Hawai'i	4
ECA744	Volcano	19.719	-155.950	Hawai'i	19
ECA746	Volcano	19.115	-155.819	Hawai'i	17
N2	Non-Hawaiian	51.450	-2.590	Great Britain	20

* indicates group was assigned based on the species tree available on CaeNDR [20250625 release]. For all other strains, group was determined directly from assignments in [47], Figure 5, and verified in the latest species tree release. Geographic information was obtained from CaeNDR.
** Island of Hawai'i (Big Island)

Table B: Details of spore preps and doses for each parasite species.

Parasite	Concentration	Low Dose	High Dose
N. parisii (ERTm1)	61,143 spores/μL	14.7μL, ~8.99 x 10^5 spores	33.1μL, ~ 20.24 x 10^5 spores
N. ironsii (ERTm5)	23,047 spores/μL	27.4μL, ~ 6.32 x 10^5 spores	63.8μL, ~ 14.70 x 10^5 spores

Doses were calibrated based on the results of infectivity assays so that ERTm1 and ERTm5 inocula were comparably infective at a given dose.

Table C: Number of hermaphrodite hosts per strain and treatment in the fitness assay.

Np = *N. parisii*; *Ni* = *N. ironsii*. *low* = low dose; *high* = high dose.

		Treatment				
**Host**	**Block**	*Control*	*Np low*	*Np high*	*Ni low*	*Ni high*
**CB4856**	1	9	9	10	9	10
**DL238**	4	9	8	8	7	9
**ECA1977**	6	10	9	9	10	10
**QX1791**	5	10	10	10	9	10
**ECA372**	2	10	8	7	8	6
**ECA705**	4	10	10	10	10	10
**ECA743**	3	10	9	10	10	9
**ECA812**	3	9	10	9	10	10
**QX1792**	6	10	10	10	10	10
**ECA2334**	5	10	10	9	10	10
**ECA347**	1	9	10	10	10	10
**ECA363**	2	10	10	10	10	10
**ECA724**	2	9	10	9	10	9
**ECA740**	3	10	9	10	10	10
**XZ1514**	4	10	7	10	9	9
**ECA1997**	4	8	7	8	7	9
**ECA730**	2	10	10	10	10	9
**ECA744**	5	10	7	8	6	7
**ECA746**	6	10	10	9	10	9
**N2***	1-6	26	30	30	29	30

*We included the strain N2 in each block to evaluate block effects. N2 was represented in full in block 1 (10 hosts per treatment), then with smaller sample sizes in the subsequent five blocks (4 hosts per treatment).

Table D: Statistical analysis of total fecundity in control and exposed conditions across host strains

A. Full model

Response	Number of offspring
Fixed effects	Host strain*Condition
Random effects	Block and Plate ID
Distribution	Gaussian

Condition = Control or Exposed, with Exposed encompassing all four exposed treatments.

B. Likelihood ratio test

Model	Fixed effects	AIC	χ^2	df	*p*
1	Host strain*Condition	10813			
			74.6	19	<0.001
2	Host strain + Condition	10849			

C. Summary of best model

Reference = CB4856, Control

Fixed Effect	Level	HostID	Coefficient ± SE	t value
Intercept			141.9 ± 24.3	5.8
Host strain	DL238	14	22.9 ± 34.2	0.7
	ECA1977	16	17.9 ± 33.8	0.5
	ECA1997	8	-11.8 ± 23.7	-0.5
	ECA2334	13	2.5 ± 22.6	0.1
	ECA347	15	21.3 ± 32.7	0.7
	ECA363	7	-7.4 ± 22.6	-0.3
	ECA372	10	15.7 ± 33.8	0.5
	ECA705	5	-18.7 ± 33.8	-0.6
	ECA724	6	-8.1 ± 34.1	-0.2
	ECA730	4	-15.1 ± 33.8	-0.4
	ECA740	3	-48.5 ± 22.6	-2.1
	ECA743	12	16.2 ± 33.8	0.5
	ECA744	19	61.1 ± 33.8	1.8
	ECA746	17	19.6 ± 22.5	0.9
	ECA812	2	-61 ± 34.1	-1.8
	N2	20	84.8 ± 30.7	2.8
	QX1791	18	29.0 ± 33.8	0.9
	QX1792	11	-3.9 ± 33.8	-0.1
	XZ1514	1	-108.8 ± 33.8	-3.2
Condition	Exposed		-91.1 ± 25.8	-3.5
Host strain:Condition	DL238:Exposed	14	28.3 ± 36.6	0.8
	ECA1977:Exposed	16	11.7 ± 36.2	0.3
	ECA1997:Exposed	8	9.9 ± 24.1	0.4
	ECA2334:Exposed	13	6.7 ± 22.7	0.3
	ECA347:Exposed	15	0.7 ± 36.4	0.0
	ECA363:Exposed	7	27.0 ± 22.7	1.2
	ECA372:Exposed	10	62.2 ± 36.4	1.7
	ECA705:Exposed	5	106.4 ± 36.1	2.9
	ECA724:Exposed	6	60.5 ± 36.5	1.7
	ECA730:Exposed	4	28.0 ± 36.2	0.8
	ECA740:Exposed	3	56.4 ± 22.7	2.5
	ECA743:Exposed	12	37.9 ± 36.2	1.0
	ECA744:Exposed	19	-20.0 ± 36.4	-0.5
	ECA746:Exposed	17	17.0 ± 22.8	0.7
	ECA812:Exposed	2	68.1 ± 36.5	1.9
	N2:Exposed	20	-14.3 ± 33.9	-0.4
	QX1791:Exposed	18	-12.4 ± 36.2	-0.3
	QX1792:Exposed	11	62.3 ± 36.1	1.7

XZ1514:Exposed	1	72.4 ± 36.2	2.0

Table E: Statistical analysis of total fecundity in control and exposed conditions across host group

A. Full model

Response	Number of offspring
Fixed effects	Host group * Condition
Random effects	Host strain and Plate ID
Distribution	Gaussian

Condition = Control or Exposed, with Exposed encompassing all four exposed treatments.
Block is not included as a random effect, because N2 is excluded from these analyses. N2 is not part of a Hawaiian group.

B. Likelihood ratio test

Model	Fixed effects	AIC	χ^2	df	*p*
1	Host group*Condition	9242			
			41.6	3	<0.001
2	Host group + Condition	9277			

C. Summary of best model

Reference = Divergent, Control

Fixed Effect	Level	Coefficient ± SE	t value
Intercept		109.4 ± 15.5	7.1
Host group	Invaded	16.9 ± 20.6	0.8
	Low	54.7 ± 20.9	2.6
	Volcano	44.4 ± 20.9	2.1
Condition	Exposed	-48.8 ± 10.9	-4.5
Host group:Condition	Invaded:Exposed	27.2 ± 11.2	2.4
	Low:Exposed	-35.7 ± 10.4	-3.4
	Volcano:Exposed	-28.3 ± 10.4	-2.7

Table F: Statistical analysis of total fecundity across treatments

A. Full model

Response	Number of offspring
Fixed effects	Host strain * Treatment
Random effects	Block
Distribution	Gaussian

Treatment = Control, *N. parisii* at low dose, *N. parisii* at high dose, *N. ironsii* at low dose, *N. ironsii* at high dose

B. Likelihood ratio test

Model	Fixed effects	AIC	χ^2	df	*p*
1	Host strain * Treatment	10820			
			161.9	76	<0.001
2	Host strain + Treatment	10830			

C. Summary of model 2 to highlight treatment main effects

Reference = Control treatment, CB4856

Fixed Effect	Level	HostID	Coefficient ± SE	t value
Intercept			119.2 ± 10.7	11.2
Treatment	*N. parisii*, low dose		-42.5 ± 4.6	-9.3
	N. parisii, high dose		-64.9 ± 4.5	-14.3
	N. ironii, low dose		-63.6 ± 4.5	-14.0
	N. ironsii, high dose		-84.5 ± 4.5	-18.6
Host strain	DL238	14	46.9 ± 14.1	3.3
	ECA1977	16	28.0 ± 13.7	2.0
	ECA1997	8	-2.6 ± 14.2	-0.2
	ECA2334	13	9.9 ± 13.8	0.7
	ECA347	15	21.6 ± 9.4	2.3
	ECA363	7	15.1 ± 13.7	1.1
	ECA372	10	64.3 ± 14.2	4.5
	ECA705	5	67.3 ± 13.8	4.9
	ECA724	6	41.3 ± 13.8	3.0
	ECA730	4	8.1 ± 13.8	0.6
	ECA740	3	-2.2 ± 13.8	-0.2
	ECA743	12	47.7 ± 13.8	3.5
	ECA744	19	50.2 ± 14.2	3.5
	ECA746	17	33.8 ± 13.7	2.5
	ECA812	2	-4.7 ± 13.8	-0.3
	N2	20	73.3 ± 9.0	8.2
	QX1791	18	21.2 ± 13.8	1.5
	QX1792	11	46.6 ± 13.6	3.4
	XZ1514	1	-50.7 ± 14.0	-3.6

Table G: Statistical analysis of change in total fecundity from control for each exposure treatment

A. Representative full model

Response	Number of offspring
Fixed effects	Host strain * Treatment
Random effects	Block
Distribution	Gaussian

Treatment = Control and EITHER *N. parisii* at low dose, *N. parisii* at high dose, *N. ironsii* at low dose, OR *N. ironsii* at high dose

B. Low dose of *N. parisii* vs. control, likelihood ratio test

Model	Fixed effects	AIC	χ^2	df	*p*
1	Host strain*Treatment	4472			
			49.6	19	<0.001
2	Host strain + Treatment	4461			

C. High dose of *N. parisii* vs. control, likelihood ratio test

Model	Fixed effects	AIC	χ^2	df	*p*
1	Host strain*Treatment	4392			
			78.1	19	<0.001
2	Host strain + Treatment	4432			

D. Low dose of *N. ironsii* vs. control, likelihood ratio test

Model	Fixed effects	AIC	χ^2	df	*p*
1	Host strain*Treatment	4404			
			52.2	19	<0.001
2	Host strain + Treatment	4418			

E. High dose of *N. ironsii* vs. control, likelihood ratio test

Model	Fixed effects	AIC	χ^2	df	*p*
1	Host strain*Treatment	4405			
			68.9	19	<0.001
2	Host strain + Treatment	4436			

Table H: Statistical analysis of total fecundity across exposed treatments

A. Full model

Response	Number of offspring
Fixed effects	Host strain * Exposure Treatment
Random effects	Block
Distribution	Gaussian

Exposure treatment = *N. parisii* at low dose, *N. parisii* at high dose, *N. ironsii* at low dose, *N. ironsii* at high dose (**Control excluded**)

B. Likelihood ratio test

Model	Fixed effects	AIC	χ^2	df	*p*
1	Host strain * Treatment	8530			
			69.6	57	0.123
2	Host strain + Treatment	8486			

C. Summary of best model

Reference = N. parisii, *low dose treatment, CB4856*

Fixed Effect	Level	HostID	Coefficient ± SE	t value
Intercept			69.8 ± 11.4	6.1
Treatment	*N. parisii*, high dose		-22.4 ± 4.2	-5.4
	N. ironii, low dose		-21.4 ± 4.2	-5.1
	N. ironsii, high dose		-42.0 ± 4.2	-10.1
Host strain	DL238	14	55.1 ± 14.8	3.7
	ECA1977	16	24.5 ± 14.4	1.7
	ECA1997	8	1.8 ± 14.8	0.1
	ECA2334	13	13.8 ± 14.3	1.0
	ECA347	15	21.9 ± 9.5	2.3
	ECA363	7	27.2 ± 14.3	1.9
	ECA372	10	85.4 ± 14.9	5.7
	ECA705	5	91 ± 14.4	6.3
	ECA724	6	59.8 ± 14.4	4.2
	ECA730	4	20.5 ± 14.3	1.4
	ECA740	3	12.5 ± 14.3	0.9
	ECA743	12	58.5 ± 14.4	4.1
	ECA744	19	45.9 ± 14.9	3.1
	ECA746	17	31.4 ± 14.4	2.2
	ECA812	2	11.6 ± 14.3	0.8
	N2	20	72.4 ± 9.2	7.9
	QX1791	18	21.2 ± 14.3	1.5
	QX1792	11	53.3 ± 14.3	3.7
	XZ1514	1	-32.8 ± 14.6	-2.2

Table I: Correlation[1] of defense[2] across exposure treatments

ρ	*N. parisii,* low dose	*N. parisii,* high dose	*N. ironsii,* low dose	*N. ironsii,* high dose
N. parisii, low dose	1.00			
N. parisii, high dose	0.65*	1.00		
N. ironsii, low dose	0.60*	0.51	1.00	
N. ironsii, high dose	0.80*	0.72*	0.63*	1.00

[1] Spearman's rank correlation coefficient ρ was calculated because defense estimates were not consistently normally distributed.

[2] Defense calculated for each host strain as the mean number of offspring per host in an exposed treatment, divided by the mean number per host in the control treatment.

[*] denotes p-value is less than alpha value of 0.008, after Bonferroni correction for six tests.

Table J: Statistical analysis of daily fecundity by host strain in the control treatment

A. Full model

Response	Number of offspring per day
Fixed effects	Host strain*Day
Random effects	Individual ID, Block
Distribution	Gaussian

B. Likelihood ratio test

Model	Fixed effects	AIC	χ^2	df	*p*
1	Host strain * Day	9237			
			425.1	76	<0.001
2	Host strain + Day	9510			

Part C excluded due to its length

Table K: Statistical analysis of daily fecundity by host group in the control treatment

A. Full model

Response	Number of offspring per day
Fixed effects	Host group*Day
Random effects	Host strain, Individual ID
Distribution	Gaussian

Block is not included as a random effect, because N2 is excluded from these analyses. N2 is not part of a Hawaiian group

B. Likelihood ratio test

Model	Fixed effects	AIC	χ^2	df	*p*
1	Host group * Day	8069			
			103.1	12	<0.001
2	Host group + Day	8184			

C. Summary of best model

Reference = Day 1, Divergent group

Fixed Effect	Level	Coefficient ± SE	t value
Intercept		7.2 ± 4.0	1.8
Day	2	54.8 ± 3.8	14.4
	3	28.5 ± 3.8	7.5
	4	0.50 ± 3.8	0.1
	5	-5.6 ± 3.8	-1.5
Host group	Invaded	5.3 ± 5.9	0.9
	Low	6.2 ± 6.3	1.0
	Volcano	5.9 ± 6.3	0.9
Host group:Day	Invaded:2	13.7 ± 5.7	2.4
	Invaded:3	-8.8 ± 5.7	-1.6
	Invaded:4	-7.8 ± 5.7	-1.4
	Invaded:5	-5.3 ± 5.7	-0.9
	Low:2	-2.2 ± 6.0	-0.4
	Low:3	27.1 ± 6.0	4.5
	Low:4	-0.3 ± 6.0	0.0
	Low:5	-6.0 ± 6.0	-1.0
	Volcano:2	2.0 ± 6.0	0.3
	Volcano:3	22.1 ± 6.0	3.7
	Volcano:4	-4.4 ± 6.0	-0.7
	Volcano:5	-6.6 ± 6.0	-1.1

Table L: Statistical analysis of daily fecundity in control and exposed conditions

A. Full model

Response	Number of offspring per day
Fixed effects	Condition*Day
Random effects	Individual ID, Host strain, Block, Plate ID
Distribution	Gaussian

Condition = Control or Exposed, with Exposed encompassing all four exposure treatments.

B. Likelihood ratio test

Model	Fixed effects	AIC	χ^2	df	*p*
1	Condition * Day	43914			
			444.0	4	<0.001
2	Condition + Day	44350			

C. Summary of best model

Reference = Control, Day 2

Fixed Effect	Level	Coefficient ± SE	t value
Intercept		75.8 ± 2.8	26.9
Day	1	-64.7 ± 1.8	-35.5
	3	-24.8 ± 1.8	-13.6
	4	-67.1 ± 1.8	-36.8
	5	-74.1 ± 1.8	-40.7
Condition	Exposed	-27.5 ± 2.3	-11.9
Condition:Day	Exposed:1	26.7 ± 2.0	13.1
	Exposed:3	-4.9 ± 2.0	-2.4
	Exposed:4	23.8 ± 2.0	11.6
	Exposed:5	26.9 ± 2.0	13.2

Table M: Linear regression of reproductive timing and defense against parasites

Fixed Effect	Coefficient ± SE	t value	*p*
Intercept	0.12 ± 0.16	0.73	0.477
Fraction early	0.78 ± 0.27	2.92	0.009

R^2 = 0.321
Adj. R^2 = 0.284

Defense = mean total offspring per host when exposed/mean total offspring per host in control, for each host strain
Reproductive timing = mean fraction of total offspring per host made on days 1 and 2 of reproduction, for each host strain, in the control treatment

Table N: Linear regression of fecundity and defense against parasites

Fixed Effect	Coefficient ± SE	t value	*p*
Intercept	0.60 ± 0.15	4.11	<0.001
Fecundity	-0.00 ± 0.00	-0.21	0.839

R^2 = 0.002
Adj. R^2 = -0.053

Defense = mean total offspring per host when exposed/mean total offspring per host in control, for each host strain
Fecundity = mean total offspring per host in control treatment, for each host strain

Table O: Replication structure for resistance assays. Columns are the replicate populations assayed for resistance, and numbers indicate the number of hermaphrodite hosts measured per replicate.

	48 hours				72 hours			
**Strain/isotype**	**1**	**2**	**3**	**4**	**1**	**2**	**3**	**4**
**CB4856**	132	50	50		35	35	35	35
**DL238**	120	51	35					
**ECA1977**	51	50	34		35	35	35	
**QX1791**	49	49	35					
**ECA372**	50	49	35					
**ECA705**	51	49	51		35	35	35	35
**ECA743**	35	49	50					
**ECA812**	49	49	31		32	34	34	34
**QX1792**	49	49	35					
**ECA2334**	50	50	50		35	36	35	37
**ECA347**	47	41	35					
**ECA363**	49	50	34		35	34	35	
**ECA724**	45	35	50		35	35	35	36
**ECA740**	49	50	50					
**XZ1514**	49	55	50					
**ECA1997**	49	49	36		36	35		
**ECA730**	49	50	35					
**ECA744**	31	55	50	33	35	35	36	
**ECA746**	53	51	50					
**N2**	85	49	35		33	35	35	

Table P: Statistical analysis of infection prevalence and load at 48 hours.

A. Full model

Response	Fraction of body area fluorescent
Fixed effects	Host strain
Random effects	Replicate, Block
Zero-inflation	Host strain
Distribution	Beta

Fluorescence indicates presence of *N. parisii*

B. Likelihood ratio test

Zero-inflation model: do host strains vary in infection prevalence?

Model	Zero-inflation term	AIC	χ^2	df	*p*
1	Host strain	-12017			
			84.5	19	<0.001
2	Intercept-only	-11970			

Conditional model: do host strains vary in infection load?

Model	Fixed effect	AIC	χ^2	df	*p*
1	Host strain	-12017			
			51.2	19	<0.001
2	Intercept-only	-12003			

C. Summary of best model

Reference = CB4856

Zero-inflation model: do host strains vary in infection prevalence?

Term	Level	HostID	Coefficient ± SE	Odds ratio [95%CI]	z value
Intercept			-1.37 ± 0.16	0.25 [0.18,0.35]	-8.4
Host strain	DL238	14	0.21 ± 0.23	1.23 [0.78,1.93]	0.9
	ECA1977	16	0.58 ± 0.25	1.78 [1.09,2.89]	2.3
	ECA1997	8	-0.20 ± 0.28	0.82 [0.47,1.42]	-0.7
	ECA2334	13	0.59 ± 0.24	1.80 [1.12,2.88]	2.4
	ECA347	15	-0.68 ± 0.33	0.51 [0.27,0.96]	-2.1
	ECA363	7	-0.14 ± 0.28	0.87 [0.50,1.50]	-0.5
	ECA372	10	-0.31 ± 0.29	0.73 [0.42,1.29]	-1.1
	ECA705	5	0.28 ± 0.25	1.32 [0.81,2.16]	1.1
	ECA724	6	0.17 ± 0.26	1.18 [0.70,1.98]	0.6
	ECA730	4	-0.15 ± 0.28	0.86 [0.50,1.48]	-0.5
	ECA740	3	-0.05 ± 0.26	0.95 [0.57,1.59]	-0.2
	ECA743	12	0.08 ± 0.27	1.09 [0.65,1.83]	0.3
	ECA744	19	-1.93 ± 0.45	0.14 [0.06,0.35]	-4.3
	ECA746	17	-0.13 ± 0.27	0.87 [0.52,1.47]	-0.5
	ECA812	2	-0.16 ± 0.28	0.85 [0.49,1.48]	-0.6
	N2	20	-0.43 ± 0.27	0.65 [0.38,1.12]	-1.6
	QX1791	18	0.22 ± 0.26	1.25 [0.75,2.08]	0.8
	QX1792	11	-0.42 ± 0.30	0.66 [0.37,1.17]	-1.4
	XZ1514	1	0.18 ± 0.25	1.20 [0.73,1.96]	0.7

Conditional model: do host strains vary in infection load?

Fixed Effect	Level	HostID	Coefficient ± SE	Odds ratio [95%CI]	z value
Intercept			-3.80 ± 0.11	0.02 [0.02,0.03]	-34.1
Host strain	DL238	14	-0.04 ± 0.11	0.96 [0.77,1.19]	-0.4
	ECA1977	16	0.28 ± 0.13	1.32 [1.03,1.69]	2.2
	ECA1997	8	-0.31 ± 0.14	0.73 [0.56,0.96]	-2.3
	ECA2334	13	0.01 ± 0.15	1.01 [0.75,1.35]	0.1
	ECA347	15	-0.23 ± 0.14	0.80 [0.60,1.05]	-1.6
	ECA363	7	0.22 ± 0.14	1.25 [0.95,1.64]	1.6
	ECA372	10	0.11 ± 0.14	1.12 [0.84,1.48]	0.8
	ECA705	5	-0.13 ± 0.14	0.88 [0.68,1.15]	-0.9
	ECA724	6	0.07 ± 0.14	1.07 [0.81,1.42]	0.5
	ECA730	4	0.00 ± 0.14	1 .00[0.75,1.32]	0.0
	ECA740	3	0.10 ± 0.14	1.11 [0.85,1.45]	0.8
	ECA743	12	-0.30 ± 0.15	0.74 [0.56,0.98]	-2.1
	ECA744	19	0.33 ± 0.13	1.40 [1.08,1.80]	2.6
	ECA746	17	0.22 ± 0.14	1.25 [0.95,1.64]	1.6
	ECA812	2	0.03 ± 0.14	1.03 [0.78,1.36]	0.2
	N2	20	0.39 ± 0.14	1.48 [1.12,1.94]	2.8
	QX1791	18	0.25 ± 0.13	1.29 [1.00,1.66]	2.0
	QX1792	11	0.14 ± 0.13	1.15 [0.88,1.49]	1.0
	XZ1514	1	0.00 ± 0.14	1 .00[0.76,1.30]	0.0

Table Q: Statistical analysis of infection prevalence and load by group at 48 hours.

A. Full model

Response	Fraction of body area fluorescent
Fixed effects	Host group
Random effects	Host strain, Replicate, Block
Zero-inflation	Host group
Distribution	Beta

Fluorescence indicates presence of *N. parisii.* N2 is excluded from these analyses because it is not part of a Hawaiian group

B. Likelihood ratio test

Zero-inflation model: do host groups vary in infection prevalence?

Model	Zero-inflation term	AIC	χ^2	df	*p*
1	Host group	-11370			
			24.5	3	<0.001
2	Intercept-only	-11351			

Conditional model: do host groups vary in infection load?

Model	Fixed effect	AIC	χ^2	df	*p*
1	Host group	-11370			
			1.8	3	0.614
2	Intercept-only	-11374			

C. Summary of best model

Reference = Divergent group

Zero-inflation model: do host groups vary in infection prevalence?

Term	Level	Coefficient ± SE	Odds ratio [95%CI]	z value
Intercept		-1.30 ± 0.08	0.27 [0.23,0.32]	-15.4
Host group	Invaded	-0.15 ± 0.13	0.86 [0.67,1.11]	-1.1
	Low	0.15 ± 0.12	1.16 [0.91,1.47]	1.2
	Volcano	-0.54 ± 0.15	0.58 [0.44,0.77]	-3.7

Conditional model: do host groups vary in infection load?

Fixed Effect	Level	Coefficient ± SE	Odds ratio [95%CI]	z value
Intercept		-3.76 ± 0.09	0.02 [0.02,0.03]	-41.0
Host group	Invaded	-0.09 ± 0.1	0.92 [0.75,1.12]	-0.8
	Low	0.06 ± 0.12	1.06 [0.84,1.34]	0.5
	Volcano	0.03 ± 0.11	1.03 [0.84,1.27]	0.3

Table R: Statistical analysis of infection load at 72 hours.

A. Full model

Response	Fraction of body area fluorescent
Fixed effects	Host strain
Random effects	Replicate, Block
Distribution	Beta

Fluorescence indicates presence of *N. parisii.* Only 5 out of 1,187 had no sign of infection (i.e., no fluorescence), so these hosts are excluded to specifically evaluate variation in load of infected hosts.

B. Likelihood ratio test

**Model**	**Fixed effect**	**AIC**	χ^2	**df**	***p***
1	Host strain	-2983			
			30.3	9	<0.001
2	Intercept-only	-2971			

C. Summary of best model

Reference = CB4856

**Fixed Effect**	**Level**	**HostID**	**Coefficient ± SE**	**Odds ratio [95%CI]**	**z value**
Intercept			-1.72 ± 0.19	0.18 [0.12,0.26]	-9.1
Host strain	ECA1977	16	-0.35 ± 0.12	0.70 [0.56,0.88]	-3.0
	ECA1997	8	-0.24 ± 0.15	0.78 [0.59,1.04]	-1.7
	ECA2334	13	-0.72 ± 0.12	0.48 [0.38,0.62]	-5.8
	ECA363	7	-0.18 ± 0.13	0.84 [0.65,1.08]	-1.4
	ECA705	5	-0.55 ± 0.12	0.58 [0.46,0.73]	-4.7
	ECA724	6	-0.45 ± 0.11	0.64 [0.51,0.80]	-3.9
	ECA744	19	-0.48 ± 0.13	0.62 [0.48,0.79]	-3.8
	ECA812	2	-0.58 ± 0.11	0.56 [0.45,0.70]	-5.2
	N2	20	-0.41 ± 0.13	0.66 [0.51,0.86]	-3.1

Table T: Statistical analysis of infection load by group at 72 hours.

A. Full model

Response	Fraction of body area fluorescent
Fixed effects	Host group
Random effects	Host strain, Replicate, Block
Distribution	Beta

Fluorescence indicates presence of *N. parisii.* Only 5 out of 1,187 had no sign of infection (i.e., no fluorescence), so these hosts are excluded to specifically evaluate variation in load of infected hosts. N2 is excluded from these analyses because it is not part of a Hawaiian group

B. Likelihood ratio test

Model	Fixed effect	AIC	χ^2	df	*p*
1	Host group	-2696			
			4.5	3	0.216
2	Intercept-only	-2697			

Table U: Linear regression of resistance and defense against parasites

A. 48 hours – 20 strains

Fixed Effect	Coefficient ± SE	t value	*p*
Intercept	0.76 ± 0.32	2.40	0.028
Load	-0.02 ± 0.13	-0.16	0.878

R^2 = 0.001
Adj. R^2 = -0.054

B. 72 hours – 10 strains

Fixed Effect	Coefficient ± SE	t value	*p*
Intercept	1.18 ± 0.43	2.74	0.026
Load	-0.04 ± 0.03	-1.10	0.302

R^2 = 0.132
Adj. R^2 = 0.024

Defense = mean total offspring per host when exposed to a low dose of ERTm1/mean total offspring per host in control, for each host strain
Resistance = mean predicted infection load, or percent body area infected, at 48 hours for each host strain. Predictions derived from the statistical model in Table S16, conditional component, to account for replicate and block effects.
