# Supplementary material for: Defense against parasites covaries with reproductive timing, not with resistance
Source: PLoS Pathog. 2026 Jul 9;22(7):e1014388. doi: 10.1371/journal.ppat.1014388 (PMC13367896; doi:10.1371/journal.ppat.1014388)
Supplement: S2 Text — (PDF) [file ppat.1014388.s009.pdf]

S2 TEXT

SUPPLEMENTAL ANALYSIS OF SURVIVAL FOR

Defense against parasites covaries with reproductive timing, not with resistance

Amanda K Gibson, Linyao Peng, Tessa Batterton, Neha Channamraju, Victoria Feist, Sarah Hesse, Anne N Janisch, and Hongyi Shui

The fitness assay provided data on both survival and fecundity of hosts. Prior studies have demonstrated that the primary fitness effect of *Nematocida* infection is on host reproduction, not survival [1,2], and this was borne out in our dataset. We therefore focused on fecundity effects in the main text. Fecundity analyses included data from hosts that died over the course of the assay, unless it was due to experimental error, so fecundity data accounted for the negative effect of mortality on lifetime fecundity.

To specifically evaluate the effect of parasite exposure on survival, we fit a Cox proportional hazards model using the package *survival* [3]. We first included parasite exposure (yes or no) as a predictor of survival, comparing control hosts to hosts in all exposed treatments. In a second model, we included treatment as a predictor, comparing hosts across all five treatments. We excluded males from these analyses.

Parasite exposure substantially reduced survival during the fitness assay (hazard ratio = 5.28, $z = 8.85$, $p < 0.001$). In the absence of parasites, 14.3% of individuals died over the course of the assay ($n = 30/209$), while in the presence of parasites, 62.9% of individuals died ($n = 515/819$). Mortality was elevated in all exposure treatments, but it was relatively low under a low dose of *N. parisii* (43.4% died, hazard ratio = 3.73, $z = 5.75$, $p < 0.001$) and relatively high with a high dose of *N. ironsii* (80.1% died, hazard ratio = 6.94, $z = 9.75$, $p < 0.001$). Most exposed individuals died in the final day of reproduction: 21% of deaths occurred on the fourth day ($n = 109/515$), and 71% occurred on the fifth and final day ($n = 367/515$). Deaths thus primarily occurred after the bulk of reproduction: 91.8% of offspring had been produced before the fourth day of reproduction, and 98.2% had been produced before the final day (S7 Fig.). We accordingly view parasite-mediated reductions in survival as having relatively minor consequences for host fitness.

LIST OF WORKS CITED

1. Troemel ER, Félix M-A, Whiteman NK, Barrière A, Ausubel FM. Microsporidia are natural intracellular parasites of the nematode *Caenorhabditis elegans*. PLoS Biol. 2008;6: e309.

2. Bubrig LT, Janisch AN, Tillet EM, Gibson AK. Contrasting parasite-mediated reductions in fitness within versus between patches of a nematode host. Evolution. 2022;76: 1556–1564. doi:10.1111/evo.14521

3. Therneau T. A Package for Survival Analysis in R. 2024. Available: https://CRAN.R-project.org/package=survival
